# Interaction of *Plasmodium falciparum* apicortin with α- and β-tubulin is critical for parasite growth and survival

**DOI:** 10.1038/s41598-021-83513-5

**Published:** 2021-02-25

**Authors:** Malabika Chakrabarti, Nishant Joshi, Geeta Kumari, Preeti Singh, Rumaisha Shoaib, Akshay Munjal, Vikash Kumar, Ankita Behl, Mohammad Abid, Swati Garg, Sonal Gupta, Shailja Singh

**Affiliations:** 1 Special Centre for Molecular Medicine, Jawaharlal Nehru University, New Delhi, 110067 India; 2 Department of Life Sciences, School of Natural Sciences, Shiv Nadar University, Gautam Budh Nagar, Noida, 201314 UP India; 3 Medicinal Chemistry Laboratory, Department of Biosciences, Jamia Millia Islamia, Jamia Nagar, New Delhi, 110025 India

**Keywords:** Biochemistry, Cell biology, Chemical biology

## Abstract

Cytoskeletal structures of Apicomplexan parasites are important for parasite replication, motility, invasion to the host cell and survival. Apicortin, an Apicomplexan specific protein appears to be a crucial factor in maintaining stability of the parasite cytoskeletal assemblies. However, the function of apicortin, in terms of interaction with microtubules still remains elusive. Herein, we have attempted to elucidate the function of *Plasmodium falciparum* apicortin by monitoring its interaction with two main components of parasite microtubular structure, α-tubulin-I and β-tubulin through in silico and in vitro studies. Further, a p25 domain binding generic drug Tamoxifen (TMX), was used to disrupt PfApicortin-tubulin interactions which led to the inhibition in growth and progression of blood stage life cycle of *P. falciparum*.

## Introduction

Malaria is a serious disease leading to significant lethal outcome worldwide^1,2^. Emergence of parasite resistance to various anti-malarials raises the concern of developing novel therapeutics to combat the disease^3,4^. In this background, identification of diverse target moieties in parasite is necessary for designing newer drug molecules. Cytoskeletal proteins are promising drug targets as destabilization of cytoskeletal structures in parasite inhibits its growth and invasion^5,6^. Identification of target proteins involved in the stabilization of cytoskeletal structures may lead to efficient methods for killing the parasite. Apicortin is a protein uniquely found to be present in Apicomplexan parasites as well as placozoan animal Trichoplax adhaerens with putative microtubule binding properties^7,8^. Characterization of *P. falciparum* apicortin in terms of interaction with parasite tubulin would elucidate its role in parasite growth and invasion.

Apicortin consists of two domains: partial p25α and doublecortin (DCX)^7^. The domains are present separately in distinct microtubule interacting proteins in oligodendrocytes, neurons and glial cells of vertebrates^9,10^. Also, the presence of p25α and DCX domains in same protein is found in the genomes of Apicomplexan organisms which had been sequenced^11^. The domain p25α is a part of tubulin polymerization promoting protein (TPPP/p25) family which is conserved among all the ciliated organisms^11^. The C terminal of TPPP protein presents p25α domain^8^. The TPPP family can be classified as long type TPPPs and short type TPPPs. In case of long type TPPPs, a stretch of 31 amino acid at the C terminal of the protein possesses high level of similarity (93%) throughout all the vertebrate species^12^. The stretch consists of microtubule binding region and a glycine rich motif,GXGXGXXR, also called as Rossmann-like motif. In case of short type TPPP, Rossman-like motif and the microtubule binding region in p25α is absent. Interestingly, apicortin present in Apicomplexan parasites possesses the specific region of p25 α domain along with microtubule binding region and Rossman like motif. Therefore, it is called as partial p25α domain^7^. However, the sequence of microtubule binding region *P. falciparum* apicortin is somewhat different from the other Apicomplexan parasites and the Rossmann-like motif is also absent^7,8^. The doublecortin domain is present at the C terminal of Apicomplexan apicortin and has similarity with DCX domains present in neuronal cells of vertebrates. It also facilitates in tubulin polymerization^13–15^.

Microtubules are present in the various cytoskeletal structures of both the asexual and sexual stages of the parasite along with other cytoskeletal proteins like actin, actin associated proteins, intermediate filaments and myosin^6,16^. Microtubular spindles are present in erythrocytic and exoerythrocytic schizonts, gametocytes, zygotes and oocysts facilitating in cell division^17,18^. Axoneme and flagella also contain micortubules formed in 9 + 2 array helping in motility of microgametes. Sub-pellicular microtubules contribute to cell shape, integrity, motility and invasion^5,6^. The main components of microtubule protofilamant are α-tubulin and β-tubulin, present in equal amount forming heteropolymeric structure. α- tubulin has two isoforms α-tubulin-I and α-tubulin-II among which α-tubulin-I is abundant in asexual stages^19,20^ whereas β-tubulin is encoded from a single gene^21^. Other than the microtubular form, tubulins are also present in unpolymerized form in the cytosol of the parasite^6^. Studying the interaction of PfApicortin with the two main components of parasite microtubule i.e. α-tubulin-I and β-tubulin would provide insight in understanding the function of PfApicortin.

In this work, we have prepared models of PfApicortin, α-tubulin-I and β-tubulin and confirmed the interaction by in silico protein–protein docking method. In order to validate the in silico data, we have cloned and expressed PfApicortin, α-tubulin-I and β-tubulin in *E.coli* and studied the interaction of PfApicortin with both the tubulins using various techniques. Further, we have used Tamoxifen, a p25 domain binder^22^ to disrupt the interaction between PfApicortin and tubulin, thereby monitoring the effect in the parasite.

## Results

### Monitoring the interaction of PfApicortin with Pfα-tubulin-I and Pfβ-tubulin through in silico docking

**Figure 1:**
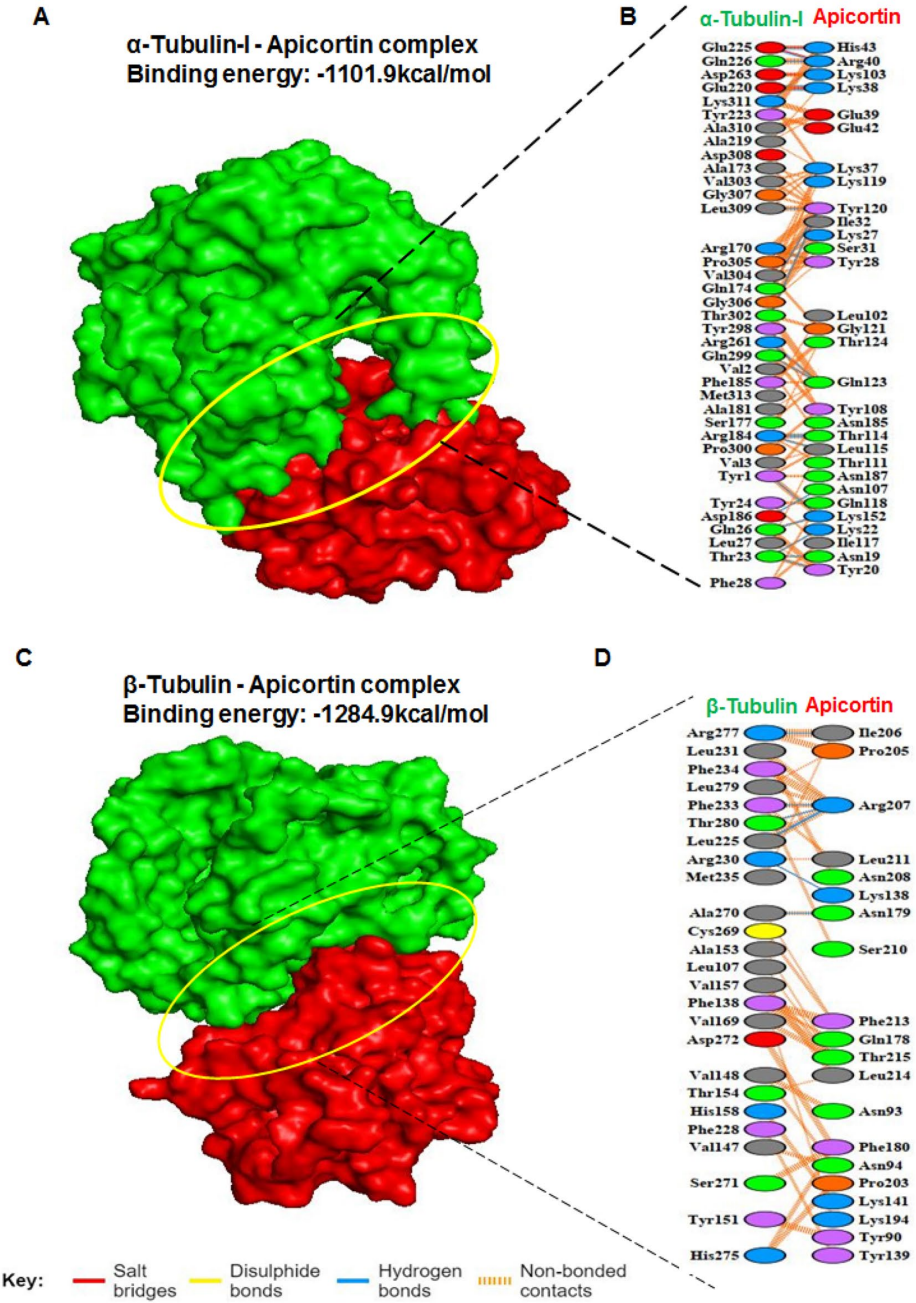
In silico docking studies showing possible binding of PfApicortin with Pfα-tubulin-I and Pfβ-tubulin. (**A**) Pfα-tubulin-I-PfApicortin complex shown in surface model with interacting residues of α-tubulin-I-Apicortin complex, (**B**) Interacting residues of Pfα-tubulin-I and PfApicortin, (**C**) Pfβ-tubulin-PfApicortin complex shown in surface model with interacting residues of Pfβ-tubulin-PfApicortin complex, (**D**) Interacting residues of Pfβ-tubulin and PfApicortin. Modes of interaction have been shown using different colours. PLIP (https://projects.biotec.tu-dresden.de/plip-web/plip), Ligplus version 2.2 (ebi.ac.uk/thornton-srv/software/LigPlus/applicence.html), Discovery Studio version 19.1.0 (https://discover.3ds.com/discovery-studio-visualizer-download) and Pymol 2.3.2 (https://pymol.org/2/) softwares were used for data analysis and generation of figures.

Putative microtubule binding property of apicortin was monitored through in silico docking of PfApicortin with Pfα-tubulin-I and Pfβ-tubulin respectively using Cluspro docking server. Structural models of the three proteins were built using ITASSER server (Supplementary Fig. S1A–C). Binding energy of PfApicortin- Pfα-tubulin-I was found − 1101.9 kcal/mol and that of PfApicortin-Pfβ-tubulin was − 1284.9 kcal/mol (Fig. 1A–D) On the basis of binding energies, it can be concluded that PfApicortin binds with α- and β-tubulin which are the major components of Plasmodium cytoskeletal structures. We also found that p25 domain residues present on the surface of apicortin (Supplementary Fig. S1A) are involved in binding with Pfα-tubulin-I and Pfβ- tubulin (Fig. 1B,D).

### Expression of Pfα-tubulin-I and Pfβ-tubulin in parasite and co-localization with PfApicortin

**Figure 2:**
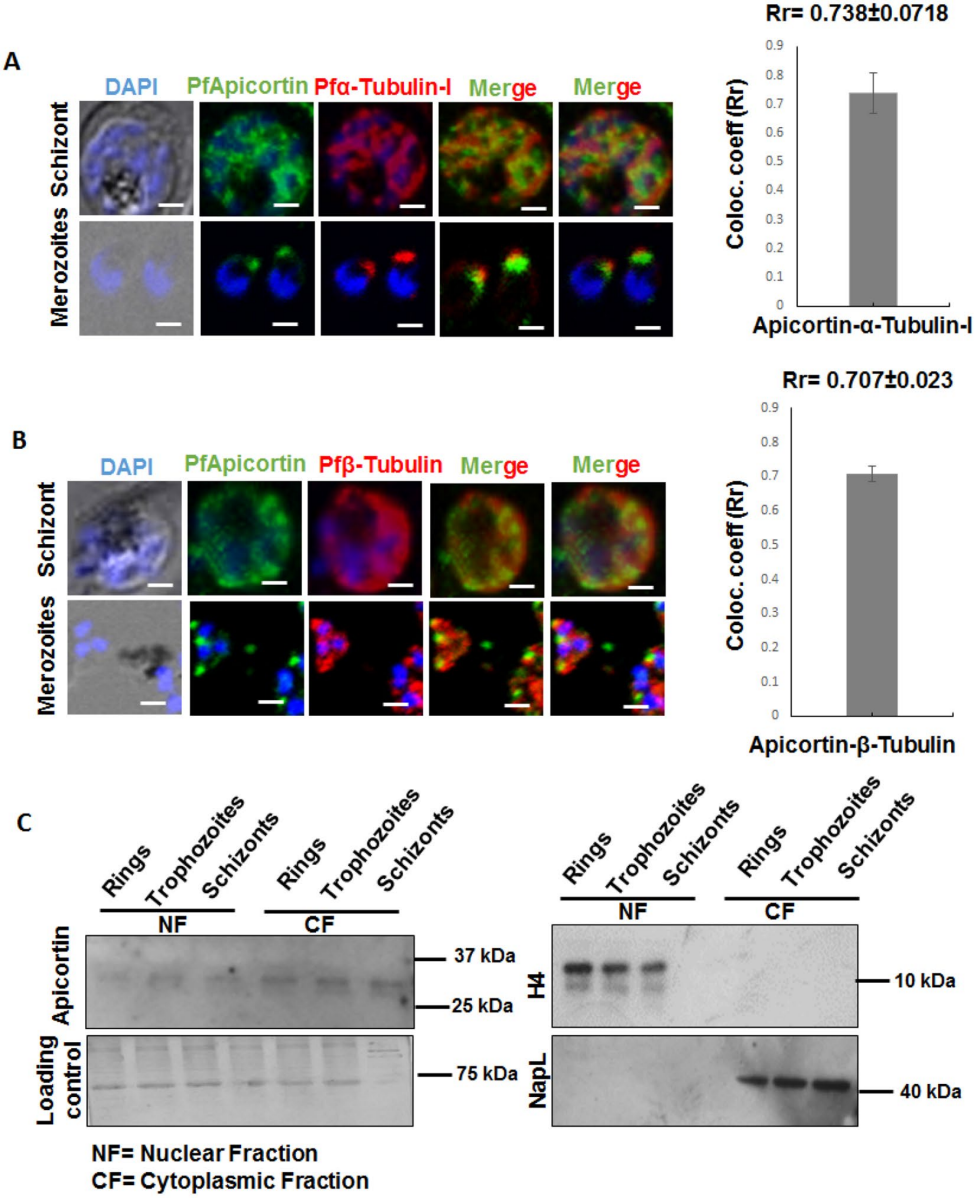
Expression and localization of Pfα-tubulin-I and Pfβ-tubulin in parasite with respect to apicortin along with localization of apicortin in subcellular fractions. (**A**) Expression of PfApicortin(green channel) and Pfα-tubulin-I(red channel) in mature schizont and merozoites along with colocalization of it with PfApicortin (merged image and graph), (**B**) Expression of PfApicortin (green channel) and Pfβ-tubulin (red channel) in mature schizont along with localization of it with PfApicortin (merged image and graph), scale bar represents distance of 5 µm. Images were analyzed with Cellsense Dimension 3 (https://www.olympus-lifescience.com/en/software/cellsens/) and ImageJ (imagej.nih.gov) softwares, (**C**) Blot showing bands of apicortin in the cytoplasmic and nuclear fractions along with the gel showing the loading control (Supplementary Fig. S9A,B) and the blots indicating the level of H4(histone) and PfNapL in nuclear and cytoplasmic fractions as controls (Supplementary Fig. S9C,D).

Localization of PfApicortin with respect to Pfα-tubulin-I and Pfβ-tubulin was monitored in trophozoites and mature schizonts (Fig. 2A,B). Localization of the tubulins was also observed in free merozoites. PfApicortin was found to localize on the surface of the parasite in subpellicular region in case of both the trophozoites and schizont (Supplementary Fig. S8, Fig. 2A,B). In the merozoites, apicortin was observed to localize at the apical end of the parasite (Fig. 2A,B). Both the tubulins were also observed to localize on parasite surface i.e. sub pellicular regions in the schizonts. In the merozoites, tubulins were also found to localize at the apical end as well as the surface (Fig. 2A,B). Higher extent of colocalization was found for apicortin- α-tubulin-I (Pearson colocalization coefficient: 0.738 ± 0.0718, Fig. 2A) as compared to β-tubulin (Pearson colocalization coefficient: 0.707 ± 0.023, Fig. 2B). Scale bar represents the distance of 5 µm. The localization of apicortin was confirmed in the nuclear and cytoplasmic extracts of the parasite by western blotting. Bands of apicortin were observed in both the fractions with lighter bands in nuclear extract indicating major occurrence of apicortin in the cytoplasm (Fig. 2C, Supplementary Fig. S9A). The coommassie stained polyacrylamide gel after transfer of the proteins prior to immunoblotting showed equal loading in all the cases (Fig. 2C, Supplementary Fig. S9B). The nuclear and cytoplasmic fractions were also probed with anti H4(histone) and anti-NapL (localized in cytoplasm) antibodies as bonafide nuclear and cytoplasmic loading controls (Fig. 2C, Supplementary Fig. S9C,D).

### Confirmation of the binding of PfApicortin with Pfα-tubulin-I and Pfβ-tubulin by immuno-precipitation, ELISA and SPR

**Figure 3:**
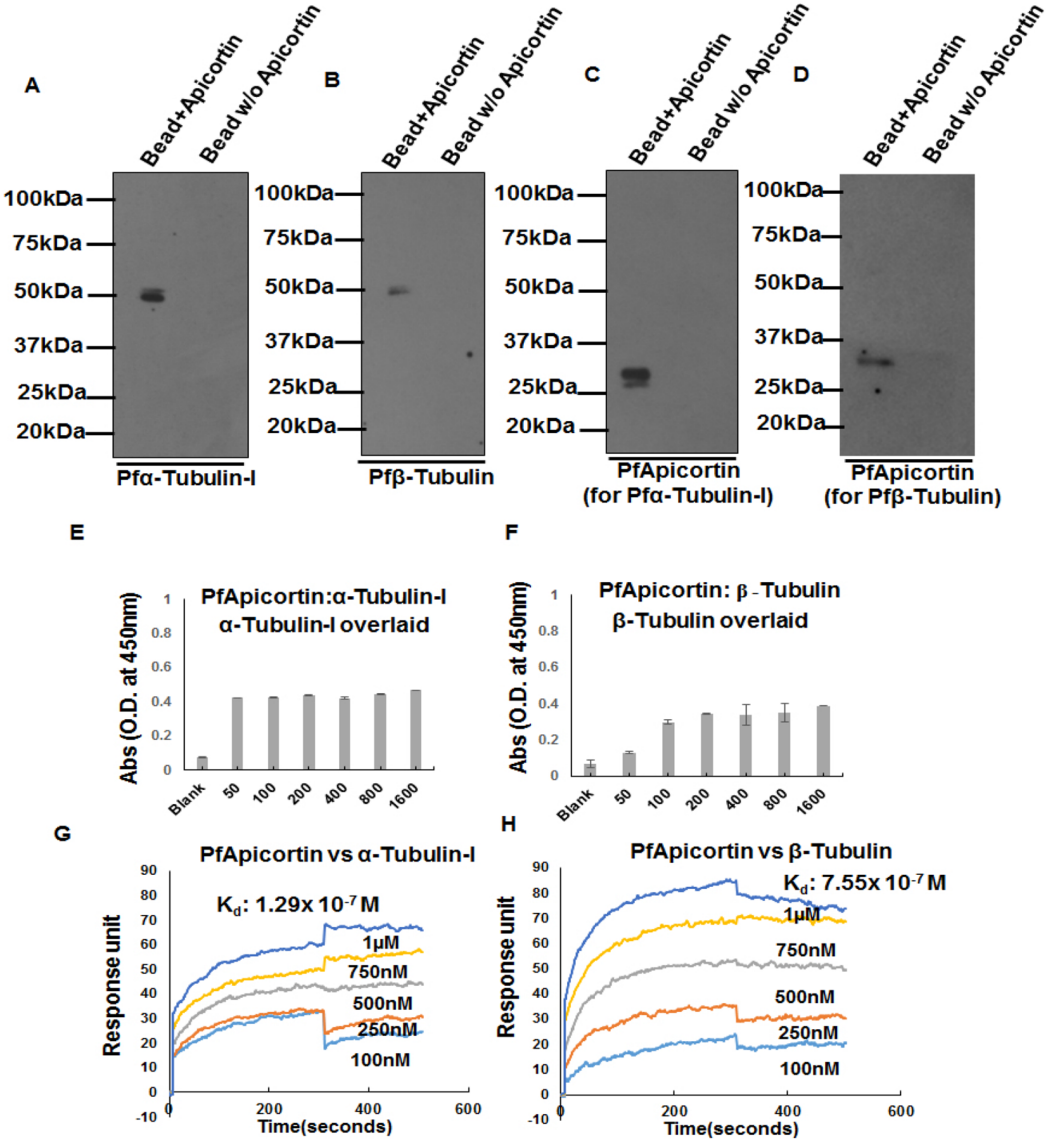
Monitoring of the binding of PfApicortin with α-tubulin-I and β-tubulin by immuno-precipitation, ELISA and SPR. (**A**) Detection of α-tubulin-I in western blotting after pulling down from parasite lysate with recombinant apicortin (Supplementary Fig. S10A), (**B**) Detection of β-tubulin in western blotting after pulling down from Pf3D7 lysate using recombinant apicortin (Supplementary Fig. S10B), (**C**) and (**D**) Confirmation of the presence of recombinant apicortin bound on the bead used for pull down assay (Supplementary Fig. S10C,D), (**E**) Graph showing indirect ELISA data showing interaction between PfApicortin and Pfα-tubulin-I with increasing titers of recombinant Pfα-tubulin-I overlaid on apicortin coated surface (x axis indicates amount of overlaid α-tubulin-I in ng), (**F**) Graph showing indirect ELISA data showing interaction between PfApicortin and Pfβ-tubulin with increasing titers of recombinant Pfβ-tubulin overlaid on apicortin coated surface (x axis indicates amount of overlaid β-tubulin in ng), (**G**) Graph showing surface plasmon resonance data where increasing concentrations of α-tubulin-I (in µM) was injected over the surface containing immobilized apicortin, (**H**) Graph showing surface plasmon resonance data where increasing concentrations of β-tubulin (in µM) was injected over the surface containing immobilized apicortin (analysis of spectra using Autolab ESPRIT kinetic evaluation software (https://www.metrohm.com/en-in/products/more-products/kei/). Data are represented as mean ± SD of at least three independent experiments.

Binding of PfApicortin with Pfα-tubulin-I and Pfβ-tubulin was confirmed by immunoprecipitation assay. Bands of Pfα-tubulin-I and Pfβ-tubulin were observed when pulled down with recombinant PfApicortin and subsequently probed with anti-tubulin antibodies (Fig. 3A,B, Supplementary Fig. S10A,B). Presence of PfApicortin in stripped blot after detection of the tubulins further confirmed the interaction between the proteins (Fig. 3C,D, Supplementary Fig. S10C,D). Presence of tubulins in input lysate was confirmed by western blotting which showed bands of α-tubulin-I and β-tubulin (Supplementary Fig. S7D,E). Further confirmation of the interaction was done by performing indirect ELISA for determining protein–protein interaction where recombinant PfApicortin was coated in protein binding plates and increasing titer of tubulins were used. Increase in absorbance was observed with increasing titers of tubulins. For α-tubulin-I, the difference in absorbance among different titers was little (Fig. 3E) whereas for β-tubulin, the absorbance increased with increasing titer of protein (Fig. 3F). In order to determine the rate of binding reaction, SPR was performed by immobilizing PfApicortin and flowing α- and β-tubulins respectively at increasing concentrations. K_d_ value of apicortin-α-tubulin-I binding was 1.2 × 10^–7^ M (Fig. 3G) and that of apicortin-β-tubulin binding was 7.5 × 10^–7^ M (Fig. 3H) indicating strong interaction between apicortin and Plasmodium tubulins.

### Binding of TMX with PfApicortin

**Figure 4:**
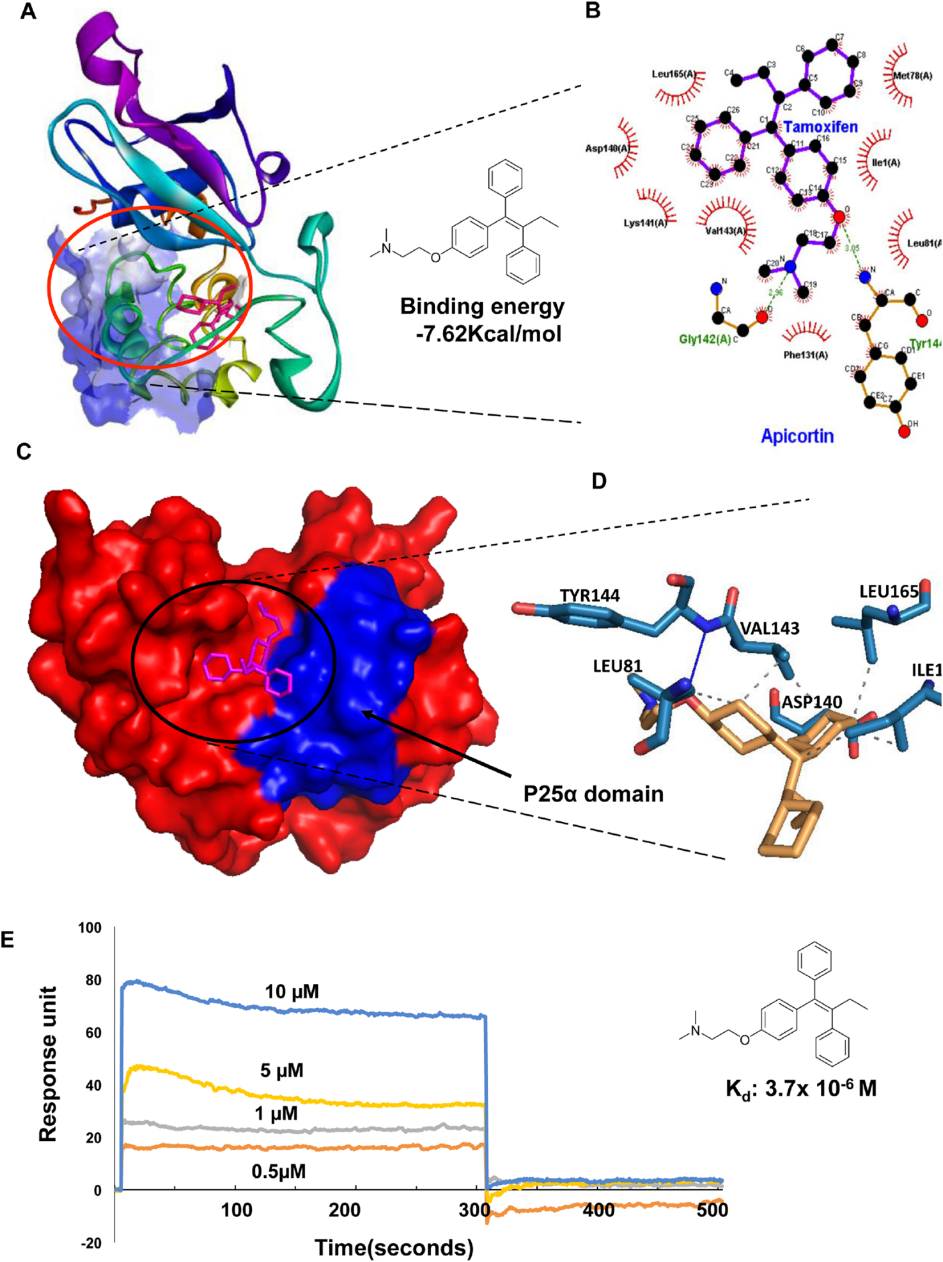
Binding of TMX with apicortin. (**A**) 3D model of apicortin-TMX complex (PLIP; https://projects.biotec.tu-dresden.de/plip-web/plip) p25α domain surface has been shown in blue scheme based on its hydrophobicity, (**B**) Ligplot analysis (ebi.ac.uk/thornton-srv/software/LigPlus/applicence.html) of apicortin-TMX complex, (**C**) 3D surface model (Pymol 2.3.2; https://pymol.org/2/) of apicortin-TMX complex with p25α domain highlighted in blue, (**D**) Interacting residues of apicortin-TMX complex, (**E**) Graph showing SPR data indicating binding of increasing concentrations of TMX (in µM) on immobilized apicortin (analysis of spectra using ESPRIT kinetic evaluation software https://www.metrohm.com/en-in/products/more-products/kei/. Data are represented as mean ± SD of at least three independent experiments.

In order to find out a small molecule candidate for binding studies with PfApicortin and obtaining proper knowledge about the function of the protein, literature was surveyed. TMX was selected to study as a candidate binder of apicortin as it binds with the p25 activator domain and disrupts the interaction of CDK5 (cyclin dependent kinase5) with p25^22^. It also possesses anti-malarial activity but the mechanism of action is not well defined^23^. Different conformations of TMX were docked on apicortin surface consisting of one chain containing multiple beta sheets and helices. The best conformations of the compound were selected based on their lowest free binding energy to the catalytic pocket (Fig. 4A). TMX showed its binding around p25α domain shown as surface (highlighted in blue) in Fig. 4C**.** Analysis of this pocket unravelled five residues including and near p25 domain (ILE1, LEU81, ASP140, VAL143 and LEU165) that are involved in interaction with the apicortin (Fig. 4B,D, Supplementary Table S1). TMX showed lower free binding energy of − 7.62 kcal/mol suggesting efficient docking (Fig. 4A,B). In the docked complex, the N atom of TMX formed one strong H-bonds with GLY142 residue of protein and O atom of TMX formed one strong H-bonds with with TYR144 of protein (2.96 Å and 3.05 Å respectively, Fig. 4D, Supplementary Table S1). Binding of TMX with apicortin was further confirmed by SPR experiment. Recombinant apicortin was immobilized on sensor SPR chip and TMX of increasing concentrations were flowed over it. Strong interaction of apicortin-TMX was observed with K_d_ value 3.7 µM (Fig. 4E). Paclitaxel and Nocodaole (tubulin binders) were used as controls for binding experiment but did not show efficient binding (Supplementary Fig. S12), with negative response units upon binding). A coumarin analogue which is a known tubulin binder^24^ was used as a negative control in this study which showed poor/nil interaction with apicortin (data not shown).

### Confirmation of TMX binding with apicortin within parasite

**Figure 5:**
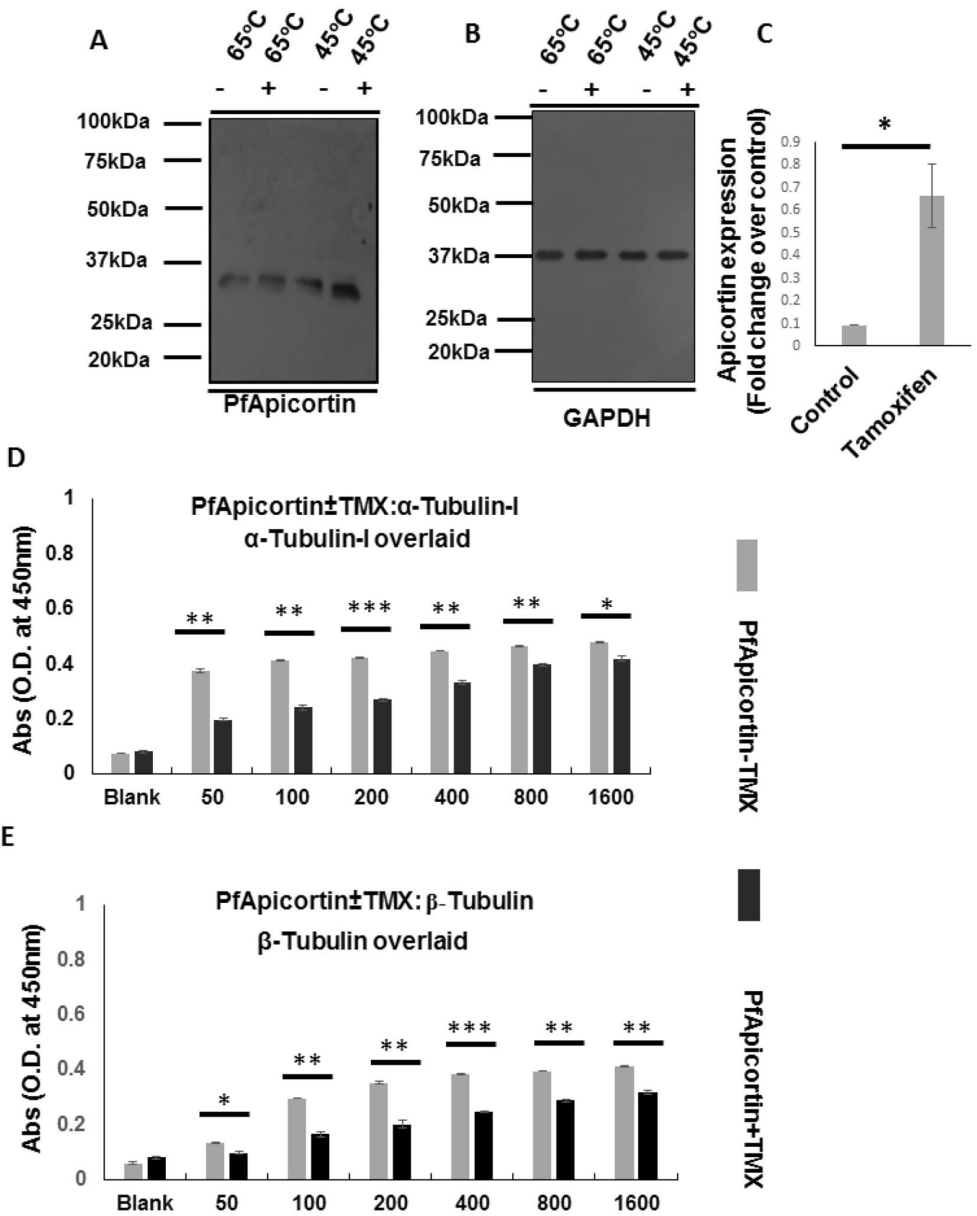
Confirmation of TMX binding with apicortin within parasite and disruption of apicortin-tubulin interaction in presence of TMX. (**A**) Western blot showing reduced band intensity of PfApicortin in thermal shift assay after treatment of parasites with TMX (presence and absence of TMX is indicated as ‘+’ and ‘−’; Supplementary Fig. S11A), (**B**) Blot showing bands of GAPDH as loading control (Supplementary Fig. S11B), (**C**) Graph showing the level of protection of PfApicortin(*p < 0.05) in thermal shift assay due to binding with TMX, (**D**) Graph showing indirect ELISA data indicating reduction in PfApicortin-α-tubulin-I interaction due to presence of TMX where recombinant α-tubulin-I was overlaid on apicortin-TMX complex coated surface (x axis indicates amount of overlaid α-tubulin-I in ng). Significant level of reduction in absorbance intensity was observed for all the titers of α- tubulin-I (50 ng, 100 ng, 400 ng, 800 ng: **p < 0.01, 200 ng:***p < 0.001, 1600 ng:*p < 0.05), (**E**) Graph showing indirect ELISA data indicating reduction in PfApicortin-β-tubulin interaction due to presence of TMX where recombinant β-tubulin was overlaid on apicortin-TMX complex coated surface (x axis indicates amount of overlaid β-tubulin in ng). Significant level of reduction in absorbance intensity was observed for all the titers of β-tubulin (100 ng, 200 ng, 800 ng, 1600 ng: **p < 0.01, 400 ng: ***p < 0.001, 50 ng: *p < 0.05). Data are represented as mean ± SD of at least three independent experiments.

The binding of TMX with PfApicortin was further confirmed by cellular thermal shift assay (CETSA) which is a method for examining ligand binding with the target protein in cell lysate or tissue extract^25^. Erythrocytes infected with *P. falciparum 3D7* trophozoites were treated with 10 µM TMX for 12 h and parasite lysate was prepared subsequently. Thermal shift assay was performed at 45 °C and 65 °C. In western blot analysis, significant difference in band intensity (Fig. 5A, S11A 5C,*p < 0.05) of PfApicortin was observed between the control (without TMX) and TMX treated samples for both the temperatures. Higher intensity of PfApicortin band in case of TMX treated sample indicates binding of TMX with parasite apicortin leading to protection of the protein from precipitation. As a loading control, GAPDH was used which showed no difference between control and the drug treated sample (Fig. 5B, Supplementary Fig. S11B).

### Disruption of apicortin-tubulin interaction in presence of TMX

TMX was used to monitor the disruption of apicortin-tubulin interaction. ELISA was performed with apicortin (pre-incubated with 10 µM TMX) coated in the microtiter plate and subsequent incubation with varying titer of tubulins was done. Decrease in absorbance intensity at 450 nm was observed in case of both the tubulins as compared to the control. In Fig. 5D,E, differences in the absorbance intensities has been shown (grey and black bars indicating absorbances of samples without TMX as well as in presence of it respectively). Significant reduction in absorbance intensities was observed for all the concentrations of both the tubulins (Fig. 5D,E) indicating possible disruption of the apicortin-tubulin interaction.

### Microtubule assembly in presence of apicortin

**Figure 6:**
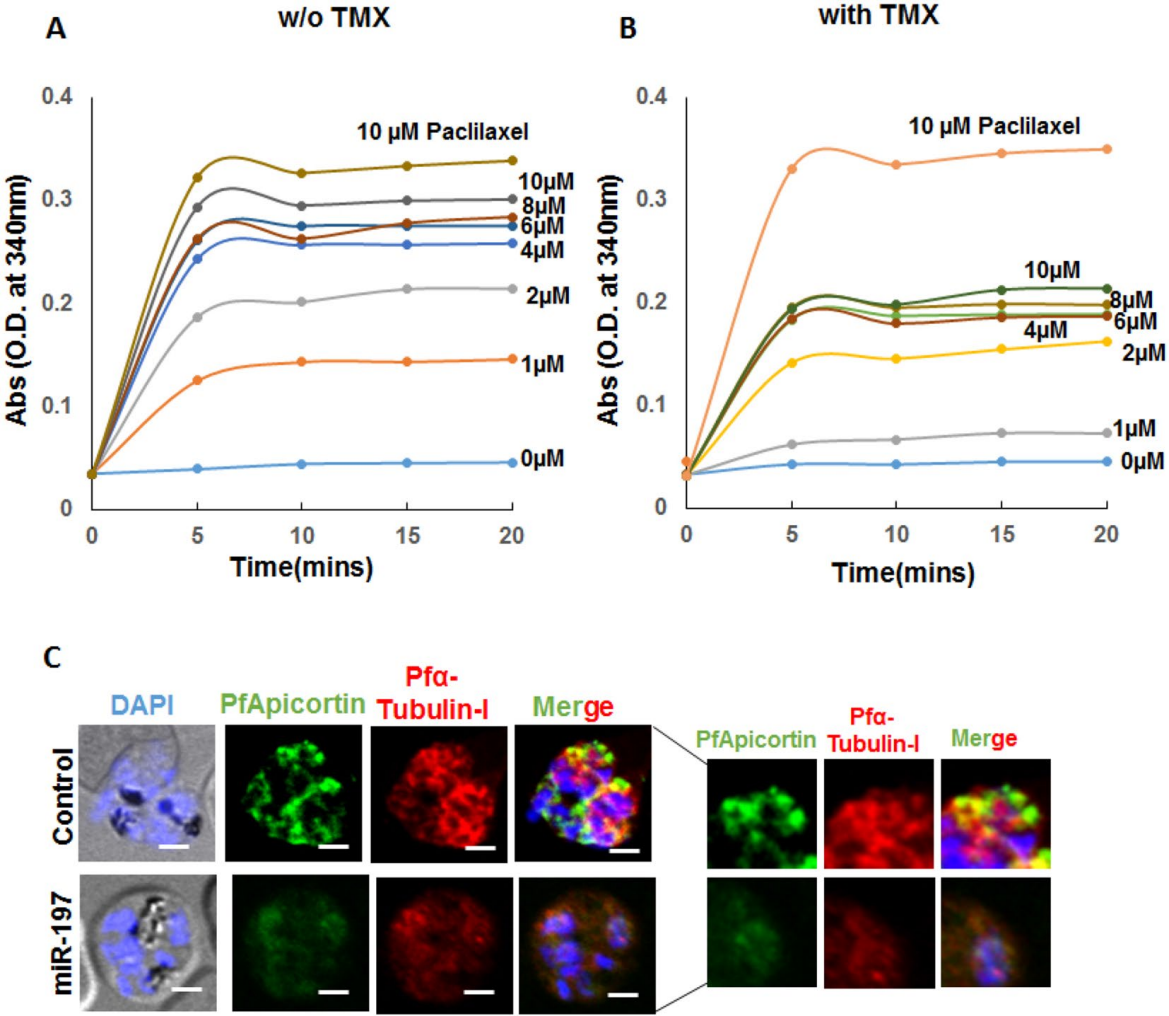
Apicortin mediated tubulin polymerization and stabilization of parasite microtubules. (**A**) Graph showing polymerization of tubulins over time in presence of increasing concentrations of apicortin, (**B**) graph showing polymerization of tubulins in presence of increasing concentrations apicortin (in µM) pre-incubated with 10 µM TMX, (**C**) reduced expression of apicortin (green channel) and diffuse staining of α-tubulin-I (red channel) in parasites infecting miR-197 loaded erythrocytes. Scale bar represents distance of 5 µm. Analysis of images performed using Cellsense Dimension 3 (https://www.olympus-lifescience.com/en/software/cellsens/) and ImageJ (imagej.nih.gov) softwares.

In order to monitor the effect of apicortin on microtubule assembly, tubulin polymerization assay was performed with equal amounts of recombinant α-tubulin-I and β-tubulin in presence of increasing concentrations of recombinant apicortin (1–10 µM). Extent of polymerization was observed by measuring the absorbance of the reaction mixture at 340 nm. With the rising concentrations of apicortin, absorbance was found to increase within a time span of 20 min (Fig. 6A). Similar experiment was performed with apicortin pre-incubated with 10 µM TMX and absorbance was monitored. Lesser extent of polymerization was observed in presence of TMX with approximately 50% reduction in absorbance in presence of 10 µM apicortin as compared to the control group (Fig. 6B). Paclitaxel (10 µM) was used as a positive control for tubulin polymerization in both the experiments. Increased level of tubulin polymerization in presence of apicortin indicates stabilization of the microtubule assembly in the parasite through apicortin-tubulin interaction. Apicortin mediated microtubular stabilization was further confirmed through downregulation of apicortin by microRNA. In our previous publication, we identified a human microRNA candidate miR-197 causing downregulation of parasite apicortin^26^. Parasites grown in miR-197 enriched erythrocytes were probed for apicortin and α-tubulin-I through immunofluorescence assay. Downregulation of apicortin was observed with decreased fluorescence intensity in green channel in the parasites infecting miRNA loaded erythrocytes. The staining of α-tubulin-I was found to be diffuse in the miRNA targeted parasites whereas clear microtubular surface structures were visible in case of the control parasite (Fig. 6C). The diffuse staining of α-tubulin-I indicates probable destabilization of the microtubule assembly as an effect of repression in apicortin expression.

### Growth inhibition and cell death of *P. falciparum* by TMX

**Figure 7:**
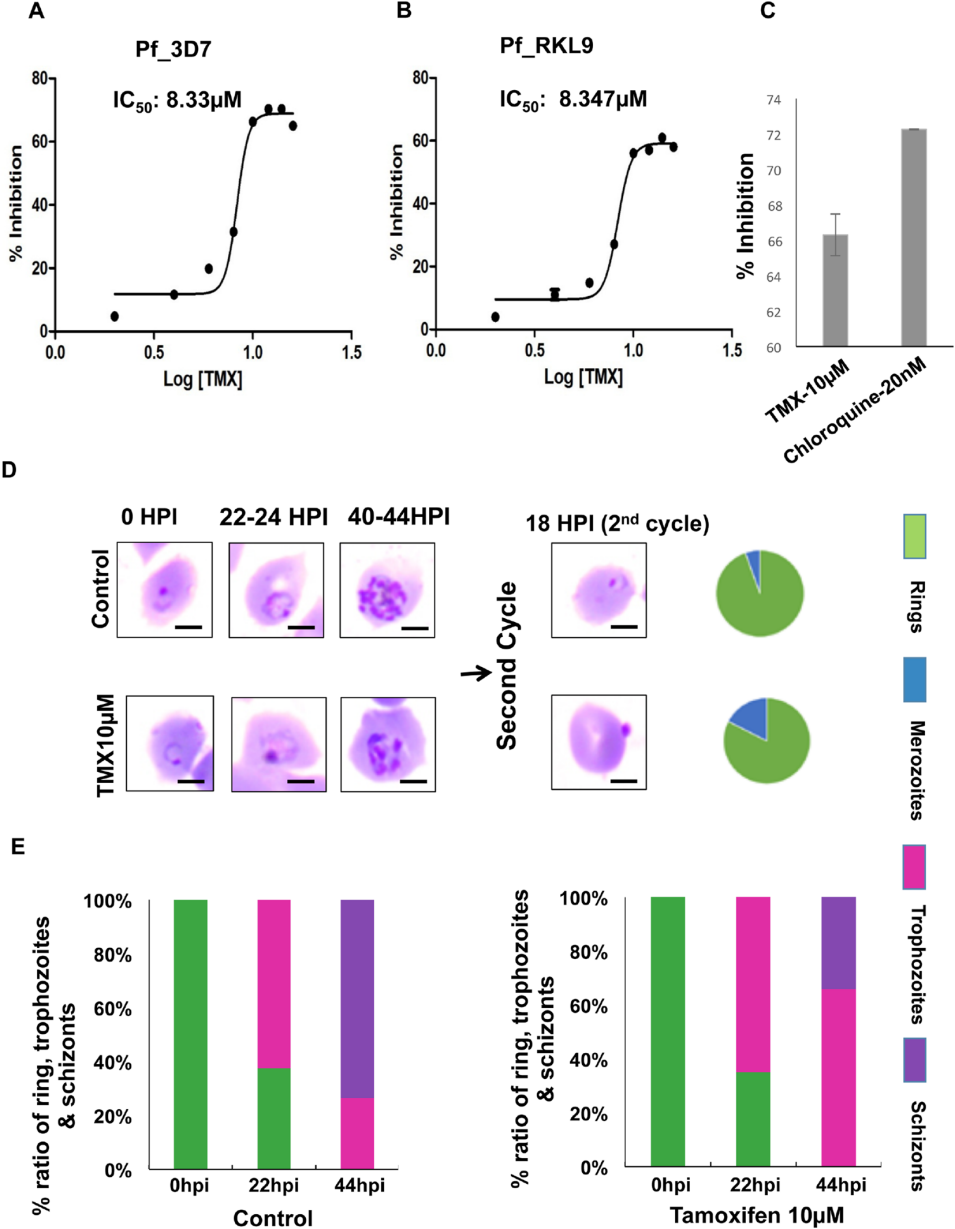
Growth inhibition and defective progression of *P. falciparum* due to TMX treatment. (**A**) Graph showing growth inhibition of Pf3D7 and (**B**) PfRKL9 in presence of TMX with IC_50_ 8.3 µM, (**C**) Graph showing percent inhibition in presence of TMX and chloroquine as positive control (**D**) Giemsa images showing hindered progression (44 hpi) and invasion (2nd cycle) of Pf3D7 with the relative percentages of ring formed and uninvaded merozoites in 2nd cycle of infection in control and TMX treated culture, scale bar represents distance of 5 µm (analysis of images by ImageJ; imagej.nih.gov) (**E**) Graph showing percent ratio of ring, trophozoite and schizonts at different time points post invasion for control and TMX treated culture. Data are represented as mean ± SD of at least three independent experiments.

Growth inhibition assay was performed by applying different concentrations of TMX in two strains of *P. falciparum*: 3D7 and RKL9 (resistant to chloroquine). Growth inhibition was observed in case of both the strains with IC_50_ value 8.3 µM (Fig. 7A,B). Progression of the parasite was also found defective in case of 10 µM TMX treated parasites as compared to the control as less number of mature schizonts were observed die to intraerythrocytic cell death at 44 h post invasion (Fig. 7D). The relative percentage of schizonts was less as compared to control at 44–46 h post invasion (Fig. 7E). In the second cycle of infection, less number of rings were formed in comparison to control and uninvaded merozoites were observed in drug treated culture (shown in pie charts, Fig. 7D). Chloroquine was used as a positive control for the growth inhibition assay with 72% growth inhibition at a concentration of 20 nM in case of *P. falciparum* 3D7 (Fig. 7C). Scale bar in Giemsa images represents the distance of 5 µm.

## Discussion

Due to the rapid emergence of malaria parasite resistance to known anti-malarials, developing newer therapeutics is essential. For that, identification of more number of targets with diverse functions is necessary. Cytoskeletal proteins play important role in parasite growth and invasion due to the formation of specialized structures like apical complex, rhoptries, microneme etc. Targeting cytoskeletal proteins (tubulins) is a prevailing strategy for designing drugs against cancer. Application of cytoskeletal protein inhibitor or microtubule binder was also found to be effective in case of malaria parasite^27^. The presence of α- and β-tubulins among different Apicomplexan protists (Supplementary Fig. S2B,C) makes them ideal component for targeting with inhibitors. The cytoskeletal assembly consists of cylindrical structures of 24 nm diameter called microtubules. Each microtubule is composed of 13 protofilaments which consists of equal amount of α- and β-tubulin. The polymerization process of microtubule is a GTP mediated process occurring at MTOC (microtubule organizing centre). Microtubule inhibitors work either by stabilizing the microtubule assembly like Taxol or disrupting the polymerization process like Nocodazole^28,29^. Therefore, tubulin polymerization and stability of microtubular structure are crucial factors for the survival of the parasite.

Several proteins are associated with the stabilization of microtubular structure. In vertebrates, proteins of MAP/Tau family are responsible for microtubule stabilization and regulation of microtubular network, recruitment of signaling proteins and execution of microtubule driven transport^30^. Similarly, Apicomplexan parasites also possess various proteins for microtubule stabilization and maintenance of specialized structures like conoid, rhoptries, microneme etc. used for invasion^17^. In Toxoplasma, two novel proteins SPM1 and SPM2 are present in the conoid to stabilize the subpellicular microtubular structure^31^. In Theileria, CLASP1 protein is responsible for the attachment of microtubules with the kinetochore and proper positioning of mitotic spindle^32^. Disruption of these proteins leads to impaired growth and invasion of the parasites. On the basis of these evidences, apicortin was selected as a target for studying further as it has microtubule binding domains, p25α and DCX with putative microtubule stabilization property. Moreover, its unique presence in Plasmodium and other Apicomplexan protists makes it a novel target to study (Supplementary Fig. S2A, S3).

In silico and experimental data suggest a strong interaction of apicortin with α- and β-tubulin of *P. falciparum*. However, SPR and ELISA data indicate somewhat stronger interaction between apicortin and α-tubulin-I as compared to β-tubulin (lower K_d_ value for Apicortin-α-tubulin-I binding in SPR). The reason behind this variation may be the refolded conformations of the proteins. In in silico experiments, an ensemble of different conformations of both the interacting proteins has been considered. In experimental conditions, fluctuation from the predicted conformations of binding may happen due to experimental conditions like temperature, pH, salt concentration etc^33^. Moreover, refolding of the proteins may give rise to a somewhat different conformation from that of the predicted binding mode. In that case, stronger interaction of apicortin and α-tubulin-I than that of apicortin and β-tubulin may result due to conformational fluctuations of apicortin as well as tubulins in course of the refolding process. Furthermore, the presence of disordered regions in proteins often influence the protein–protein interactions^33^. Plasmodium apicortin possesses a disordered region at its N terminal^8^. Presence of the disordered extension often set limitations in in silico prediction of binding of two proteins^33^. Refolding of the disordered extension might influence the apicortin-tubulin interaction which is evident as a varied binding affinity of apicortin with the tubulins in comparison to the in silico predictions. As α- tubulin-I is abundant in asexual stage of the parasite, disruption of its interaction with apicortin might affect the invasion and progression of the parasite in erythrocytes.

The interaction of Plasmodium apicortin with parasite microtubule components α- and β-tubulins is an interesting area which needs to be explored further. Individual domains of apicortin are known to stabilize microtubules or cause polymerization of tubulins to different structures in brain cells^7^. Orthologue of apicortin in *T. gondii* known as TgDCX was found to stabilize the tubulin dimers of the microtubular structures present in the conoid of the parasite. Loss of TgDCX through knock out resulted in abnormal conoid structure along with loss of tubulin polymers from the conoid fibers. The growth and invasion of the parasite were also hindered^14^. In the previous work, we identified human microRNA candidates causing reduction in apicortin expression^26^. The growth and invasion of the malaria parasite was stalled in response to the downregulation of apicortin. In this work, we investigated further checking for the interaction of apicortin with plasmodium tubulins present in erythrocytic stages. Interaction of apicortin with α-tubulin-I and β-tubulin was confirmed by in silico docking studies as well as experimental techniques such as immunoprecipitation, ELISA and surface plasmon resonance. In order to understand the effect of apicortin on microtubular assembly stabilization, polymerization assay was performed in presence of apicortin which showed higher extent of tubulin polymerization with the rising concentrations of apicortin (Fig. 6A). The polymerization process was also affected in presence of TMX, an apicortin binder used in this study (Fig. 6B). Furthermore, downregulation of apicortin by miR-197 resulted in loss of the organized structure of tubulin (Fig. 6C). On the basis of these results, it can be inferred that Plasmodium apicortin plays critical role in microtubular structure stabilization through the interaction with parasite tubulins.

In order to find out small molecule inhibitors, binders of individual domains of apicortin were searched. TMX was selected as it has p25 domain binding property as well as anti-malarial activity^22,34^. In earlier studies, the activity of TMX against *P. falciparum* and *P. berghei* had been shown^34–38^. Although the activity of TMX was reported at higher concentration^38^, other studies documented its inhibitory effect at 10 µM concentration^34,35,37^. In an ex vivo cell line infection model with *P. berghei* infection^36^, TMX showed IC_50_ value at 4 µM. In our work, the IC_50_ of TMX was determined as 8.3 µM and the binding studies of apicortin with TMX showed effective result at a TMX concentration of 10 µM supporting the previous studies. Moreover, docking studies, cellular thermal shift assay and SPR experiment confirmed the binding of TMX with apicortin. From ELISA and polymerization assay data (Figs. 5D,E, 6B), it can be inferred that the binding of TMX with apicortin disrupted the interaction of apicortin and both the tubulins by hindering tubulin assembly which led to the death of the parasite (Fig. 7). The possible reason behind TMX mediated disruption of apicortin-tubulin interaction might be the conformational change of apicortin due to the binding of TMX at p25α domain. Similar molecules can be designed to develop novel anti-malarial therapeutics targeting an important and unique parasite protein. Also, any compound binding with the DCX domain might inhibit the apicortin-tubulin interaction in this way and serve as a probable anti-malarial therapeutic agent.

## Summary

**Figure 8:**
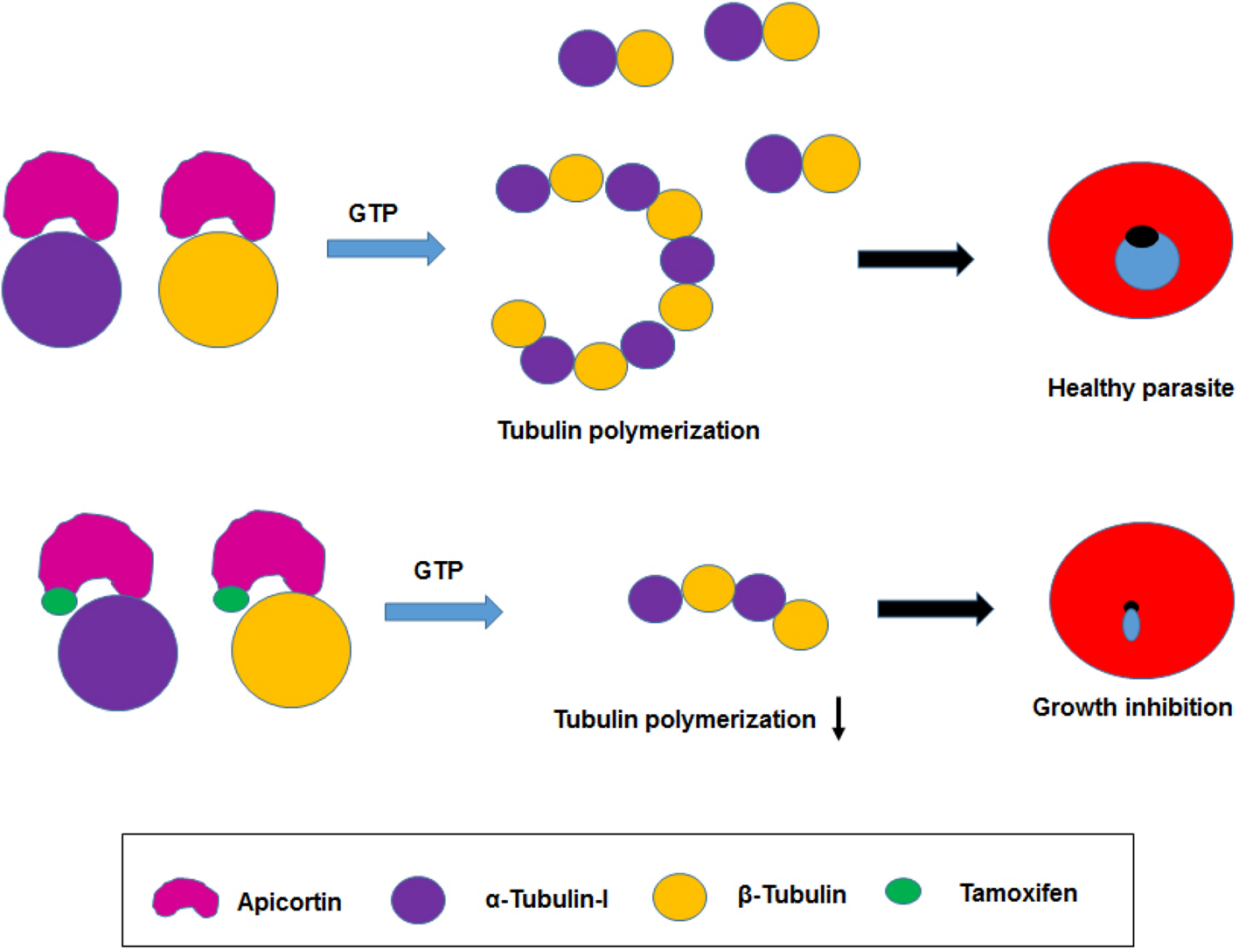
Schematic showing interaction of apicortin with tubulins in absence and presence of TMX affecting parasite growth.

It can be concluded from the current study that apicortin, an Apicomplexan parasite specific protein interacts with *P. falciparum* α-tubulin-I and β-tubulin inducing tubulin polymerization and stabilization of microtubules. Disruption of this interaction hinders the growth and progression of the asexual blood stage parasites (Fig. 8).

## Materials and methods

### Cloning, expression and purification of PfApicortin, Pfα-tubulin-I and Pfβ-tubulin

PfApicortin (NCBI accession. XM_002808649.1), Pfα-tubulin-I (NCBI accession. XM_001351875.1) and Pfβ-tubulin (NCBI accession. XM_001347333.1) were cloned (Supplementary Figs. S4A,C, S5A) in pET28a + vector digested with SnaBII (NEB, MA, USA) and subsequently dephosphorylated. PfApicortin was expressed in *E.coli* codon plus strain (Supplementary Fig. S4B) with an induction of 1 mM IPTG (Sigma-aldrich) at 37 °C for 6 h with shaking of 150 RPM. Both the tubulins were expressed in *E. coli* BL21 strain (Supplementary Figs. S4D, S5B) with an induction of 1 mM IPTG at 25 °C overnight at shaking of 150 RPM. All the proteins were solubilized from inclusion bodies using urea buffer composed of 8 M urea, 20 mM Tris, 250 mM NaCl (Sigma, USA) with pH 8.0. Solubilized proteins were purified by Ni–NTA (Qiagen, Hilden, Germany) affinity chromatography. Apicortin was eluted using 50 mM and 85 mM imidazole solution prepared in urea buffer (Supplementary Fig. S5C). α-tubulin-I was eluted using 50 mM, 100 mM, 150 mM and 200 mM imidazole solution (Supplementary Fig. S6A) and β-tubulin was eluted using 25 mM and 50 mM imidazole solution (Supplementary Fig. S6B) prepared in urea buffer. Eluted fractions were refolded using refolding buffer composed of 100 mM Tris, 20% Glycerol, 250 mM L-Arg, 1 mM EDTA, 1 mM GSG and 0.5 mM GSSG with pH 8^39^. Refolded protein was reconstituted in PBS (pH 7.4) through buffer exchange method and concentrated using Centricon tubes with 10 kDa cut-off (Merck, Germany) (Supplementary Fig. S7A–C) for performing further experiments.

### Antibody generation of the proteins

The purified proteins were injected into BALB/c mice (6 weeks, female) and they were bled twice followed by the first and second booster. Tubulins were also injected in white albino rabbits in order to raise anti-rabbit sera for tubulins. Antibodies of apicortin and tubulins were validated by western blot and immunofluorescence assay published in our previous articles^24,26^. All the experimental steps regarding immunization of mouse and rabbit were performed according to CPCSEA guidelines and approved by Institutional Animal Ethics Committee (IAEC), Jawaharlal Nehru University, New Delhi.

### Parasite culture and growth inhibition assay

Malaria parasite *P. falciparum* strain 3D7 and chloroquine resistant strain RKL9 were cultured using O + packed erythrocytes using complete RPMI 1640 supplemented with AlbumaxII (Gibco, USA), hypoxanthine (Sigma-Aldrich, MA, USA) and gentamycin (Gibco, USA) in 37 °C incubator containing a mixed gas composition of 2% O_2_, 5.5% CO_2_ and 92.5% N_2_. Parasites were synchronized using 5% Sorbitol (Sigma-Aldrich, USA) in two consecutive cycles. Assay was set up with tightly synchronized ring stage parasite culture with 1% parasitemia and 2% hematocrit in 96 well microtiter plates. Different concentrations of TMX (Cayman Chemical, USA) ranging from 2 µM to 20 µM (2 µM, 4 µM, 6 µM, 8 µM, 10 µM, 12 µM, 14 µM, 16 µM, 18 µM, 20 µM) were added to the parasites and the plates were incubated in 37 °C incubator containing a mixed gas composition mentioned earlier. Growth and invasion of the parasites were monitored from Giemsa (Sigma, USA) stained smears prepared at different time points. Invasion of the parasites were monitored using SYBR green dye assay at 48 h post invasion. Fluorescence was measured using microtiter plate reader (Varioskan Flash, Thermo, USA) with excitation at 485 nm and emission at 530 nm. Calculation of growth inhibition (% Inhibition) was done using the following formula: % Inhibition = [1 − % Fluorescence intensity (Treatment)/% Fluorescence intensity (Control)] × 100. IC_50_ was determined by plotting the respective percent inhibitions of different concentrations of the drug using Graphpad Prism5 (CA, USA) software in non-linear regression mode.

### Preparation of cytoplasmic and nuclear extracts

Fractionation of *P. falciparum* cell lysate was performed following previously published protocols^40,41^. Briefly, sorbitol synchronized *P. falciparum* 3D7 culture of ring trophozoite and schizont stages were pelleted down and lysed with the help of lysis buffer composed of 20 mM HEPES, 10 mM KCl, 1 mM EDTA, 1 mM DTT (Sigma Aldrich, USA) along with protease inhibitor (Thermo, USA). The cells were incubated in the buffer for 5 min in ice followed by centrifugation at 2500*g* at 4 °C for 10 min. The cytoplasmic extract was present in the supernatant obtained which was separated and stored at − 20 °C. The pellets were further washed twice with the lysis buffer and incubated in that buffer for 30 min at rotating condition in 4 °C. The suspension was centrifuged at 12000*g* for 30 min and the nuclear extract was obtained in the supernatant. The extracts were used for western blotting after protein estimation by Bicinchoninic acid assay kit (G Biosciences,India) following the manufacturer’s protocol.

### Immunoprecipitation

Plasmodium tubulins (α and β) were detected by pulling down with purified recombinant apicortin from *P. falciparum* cell lysate. Briefly, recombinant PfApicortin (50 µg) was bound with Ni–NTA beads (50 µl packed) followed by washing of the beads with PBS to remove unbound protein. Parasite lysate (100 µg) was incubated with the beads at 4 °C overnight and beads were further washed to remove unbound proteins. The beads were finally boiled after adding 5 × loading dye and the supernatant was loaded in polyacrylamide gel (12%). Presence of α- and β-tubulin was detected through western blotting using anti-mouse polyclonal antibodies raised against the proteins. Ni–NTA beads incubated with PBS only (without recombinant apicortin) were used as control. The input lysate incubated with both types of beads was run in gel and immunoblotted to detect the presence of α- and β-tubulin as input controls (Supplementary Fig. S7D,E). Estimation of the proteins were performed using Bicinchoninic acid assay kit (G Biosciences, India) following the manufacturer’s protocol.

### ELISA

Recombinant PfApicortin (100 ng) was coated using bicarbonate buffer in 96 well microtiter plates (Nunc, Thermo, MA, USA) at 4 °C overnight. Unbound protein was washed with PBS and blocking was done with 3% BSA (VWR, Pennsylvania, USA). Recombinant α- and β-tubulin of different titer (50 ng, 100 ng, 200 ng, 400 ng, 800 ng and 1600 ng) was incubated for 1 h at 37 °C with the coated apicortin. Unbound protein was washed and incubation with anti-mouse antibody for tubulin (1:10,000) was done at 37 °C for 1 h followed by washing with PBS and incubation with HRP conjugated anti mouse secondary antibody (1:30,000). After washing, TMB detection reagent (Himedia, India) was added and the reaction was stopped using stop buffer (1 N H_2_SO_4_). Absorbance was detected at 450 nm in microplate reader (Varioskan, Thermo, USA).

### CETSA

Thermal shift assay was performed in order to confirm the binding of TMX with apicortin inducing thermal stabilization at increased temperature^25^**.** The assay was performed following formerly published protocols in studies of screening of ligands**.** Screening of ligand binding through this method requires the use of recombinant target protein. However, methods have been developed which uses cell lysate for the purpose of checking ligand binding^42^. Briefly, cells are incubated with different compounds followed by the preparation of cell lysate. The lysate is heated at different temperatures and centrifuged subsequently in order to segregate precipitated proteins. The soluble fraction of the proteins present in the lysate supernatant is monitored through western blotting. In order to perform this assay, *P. falciparum* 3D7 culture with 10% parasitemia and 2% hematocrit was incubated with 10 µM TMX (Cayman Chemical, USA) overnight at 37 °C. The culture was harvested, treated with 0.1% saponin (Sigma, USA) for erythrocyte lysis and the parasite pellet obtained was dissolved in RIPA buffer (G-Biosciences, India) in order to prepare parasite lysate. The control and drug treated lysates were heated at 45 °C and 65 °C for 3 min and subsequently cooled down for 5 min at room temperature. The samples were centrifuged at 13,000 RPM at 4 °C for 15 min and run in 12% polyacrylamide gel. Level of apicortin in lysates was checked by subsequent western blotting.

### Western blotting

Samples derived after immune-precipitation and CETSA experiments were run in 12% SDS–polyacrylamide gel and the proteins in gel were subsequently transferred to nitrocellulose membrane (Biorad, USA). Blocking was done in 5% skim milk (Himedia, India) for 2 h at room temperature and the blot was incubated with the primary antibody (anti-apicortin and anti tubulin, 1:5000) overnight at 4 °C. The blot was further incubated with HRP conjugated anti-mouse secondary antibody for 1 h at room temperature (1:5000, Sigma-Aldrich) after washing with PBST. The blot was washed further and antibody binding was detected using Enhanced Chemiluminescence Kit (Biorad, USA).

### Immunofluorescence assay

Smears of infected erythrocytes were prepared and fixation was done by dipping the smears in chilled methanol for 20 min at − 20 °C. Smears were dried and blocked in 3% BSA (VWR, Pennsylvania, USA) solution in PBS overnight at 4 °C. Smears were incubated with anti-apicortin and anti-tubulin primary antibody for 2 h at room temperature. Further incubation with secondary antibody (conjugated with Alexa488/546, Invitrogen, USA) was done at room temperature for 1 h followed by washing with PBS. Slides containing smears were mounted using DAPI-antifade (Invitrogen, CA, USA). Images were captured in confocal microscope at 100 × magnification (Olympus Corporation, Tokyo, Japan) and analysed in CellSense Dimension 3 and ImageJ software.

### Surface Plasmon Resonance (SPR) spectroscopy

Surface Plasmon resonance spectroscopy was performed to monitor the interaction of apicortin and tubulin as well as apicortin and TMX. Immobilization of Apicortin (12 µM) was done on the activated (through amine coupling) gold sensor chip. Different concentrations of α-tubulin-I (100 nM, 250 nM, 500 nM, 750 nM, 1 µM), β-tubulin (100 nM, 250 nM, 500 nM, 750 nM, 1 µM) and TMX (0.5 µM, 1 µM, 5 µM, 10 µM) were injected over the chip surface in order to monitor the interaction both for the association and dissociation throughout 500 s using the Auto LAB ESPRIT SPR instrument (Kinetic Evaluation Instruments BV, The Netherlands) with an open cuvette system along with electrochemical resonance facility. PBS was used for immobilization and binding solution. The surface was regenerated with 50 mM NaOH. Data were analyzed using Auto Lab ESPRIT Kinetic evaluation software.

### Tubulin polymerization assay

Polymerization of recombinant tubulins were performed following the protocol published previously in studying microtubule associated proteins^43,44^. Polymerization reaction was set up with 10 µg of recombinant α- and β-tubulin and increasing concentrations of recombinant apicortin (1 µM, 2 µM, 4 µM, 6 µM, 8 µM and 10 µM) in presence of 1 mM GTP (Thermo, USA) and assembly buffer consisting of 100 mM PIPES, 1 mM EGTA and 1 mM MgCl_2_ (Sigma-Aldrich, USA) with pH 6.8. The reaction mixture was incubated at 37 °C and absorbance was measured at different time points at 340 nm in UV–Vis spectrophotometer (Cary5000, Agilent technologies, USA). The assay was also set up in different concentrations of recombinant apicortin pre-incubated with 10 µM TMX (Cayman Chemical). One reaction was set up in presence of Paclitaxel (Sigma-Aldrich, USA) as a positive control of tubulin polymerization.

### Loading of erythrocytes with miR-197 mimic

Packed erythrocytes were loaded with miR-197 mimic following the protocol mentioned in our previous publication^26^. Erythrocytes were lysed and filled with the cargo mimic followed by resealing and storage at 4 °C for growth inhibition assay.

### In silico experiments

Ab initio modelling of proteins was performed using I-TASSER server^45^. Protein sequences of *Plasmodium falciparum* apicortin (PF3D7_0517800), *Plasmodium falciparum* α-tubulin-I (PF3D7_0903700) and *Plasmodium falciparum* β-tubulin (PF3D7_1008700) were obtained from Uniprot^46^ and also confirmed from the cloned sequence. Chemical structures of TMX was prepared through ChemSketch^47^. Protein and Ligand structures were optimized using Swiss PDBviewer version 4.1.0 and ChemBio3D ultra 12.0 respectively^48^. Autodock version 4.2 was utilized to predict interaction of *Plasmodium falciparum* apicortin with TMX^49^. ClusPro server was used for protein–protein docking to predict interaction of *Plasmodium falciparum* apicortin with α-tubulin-I and β-tubulin. PLIP, Ligplus version 2.2, Discovery Studio version 19.1.0 and Pymol 2.3.2 softwares were used for further analysis and visualization of docking results^50–52^. Similarity of PfApicortin p25 domain and tubulins with other Apicomplexan parasites was determined by construction of phylogenetic tree using MEGAX alignment editor and CLUSTAL W algorithm.

### Statistical analysis

p values were calculated applying Student’s t-test wherever applicable. Results were represented as mean ± SD of at least three independent experiments.

### Compliance with ethical standards

All the experiments were carried out in accordance with the guidelines and regulations of Jawaharlal Nehru University and approved by institutional IBSC committee. Animal handling and sera generation were performed as per CPCSEA guidelines and approved by the Institutional Animal Ethics Committee (IAEC), Jawaharlal Nehru University, New Delhi. All the steps of animal handling and sera generation were performed in compliance with the ARRIVE guidelines.

## Supplementary information

Supplementary information.

## Abbreviations

DCX: Doublecortin domain
TMX: Tamoxifen
CETSA: Cellular Thermal Shift Assay
