# Supplementary material for: Interaction of Plasmodium falciparum apicortin with α- and β-tubulin is critical for parasite growth and survival
Source: Sci Rep. 2021 Feb 25;11:4688. doi: 10.1038/s41598-021-83513-5 (PMC7907060; doi:10.1038/s41598-021-83513-5)
Supplement: Supplementary file 1 — Supplementary information. [file 41598_2021_83513_MOESM1_ESM.docx]

**Supplementary Information**

**Supplementary Information**

**Interaction of *Plasmodium falciparum* apicortin with α- and β- tubulin is critical for parasite growth and survival**

**Malabika Chakrabarti^1^, Nishant Joshi^2,^, Geeta Kumari^1^, Preeti Singh^1^, Rumaisha Shoaib^3^, Akshay Munjal^1^, Vikash Kumar^1^, Ankita Behl^1^, Mohammad Abid^3^, Sonal Gupta^1^, Swati Garg^1^, Shailja Singh^1,2,^***

1. Special Centre for Molecular Medicine, Jawaharlal Nehru University, New Delhi, India

2. Department of Life Sciences, School of Natural Sciences, Shiv Nadar University, Gautam Budh Nagar, Noida, UP, India

3. Medicinal Chemistry Laboratory, Department of Biosciences, Jamia Millia Islamia, Jamia Nagar, New Delhi, 110025, India

*Corresponding information: Shailja Singh, Ph.D.

Email: [shailja.jnu@gmail.com](mailto:shailja.jnu@gmail.com), Phone (O): +91-011-2663801, Ext: 201


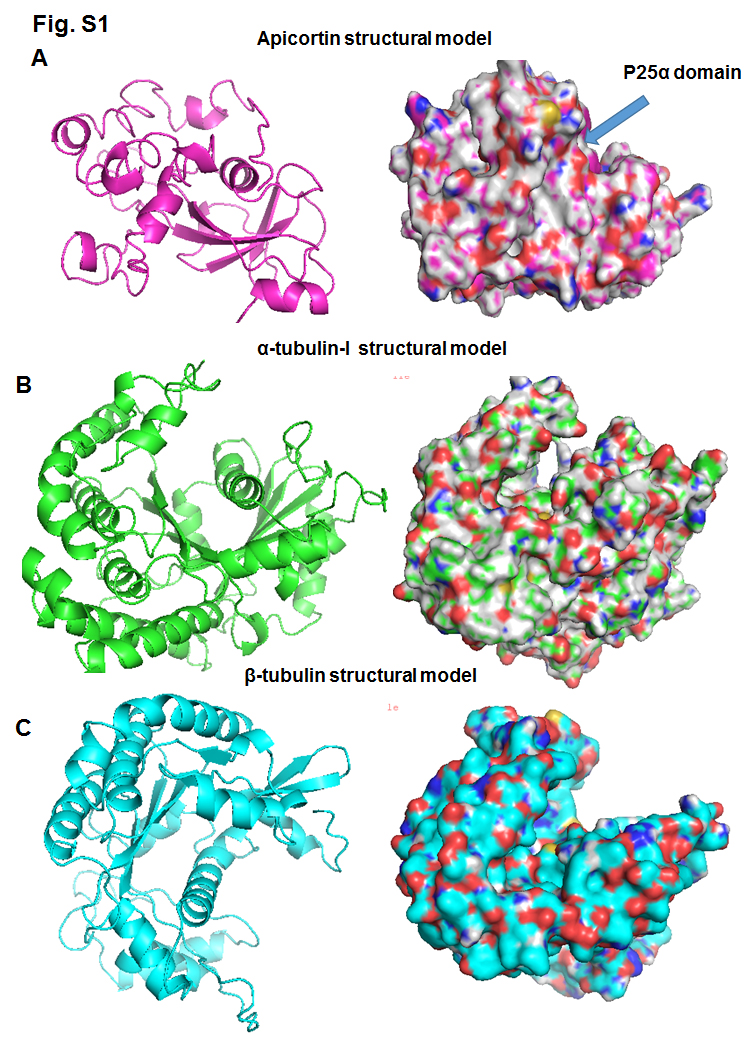

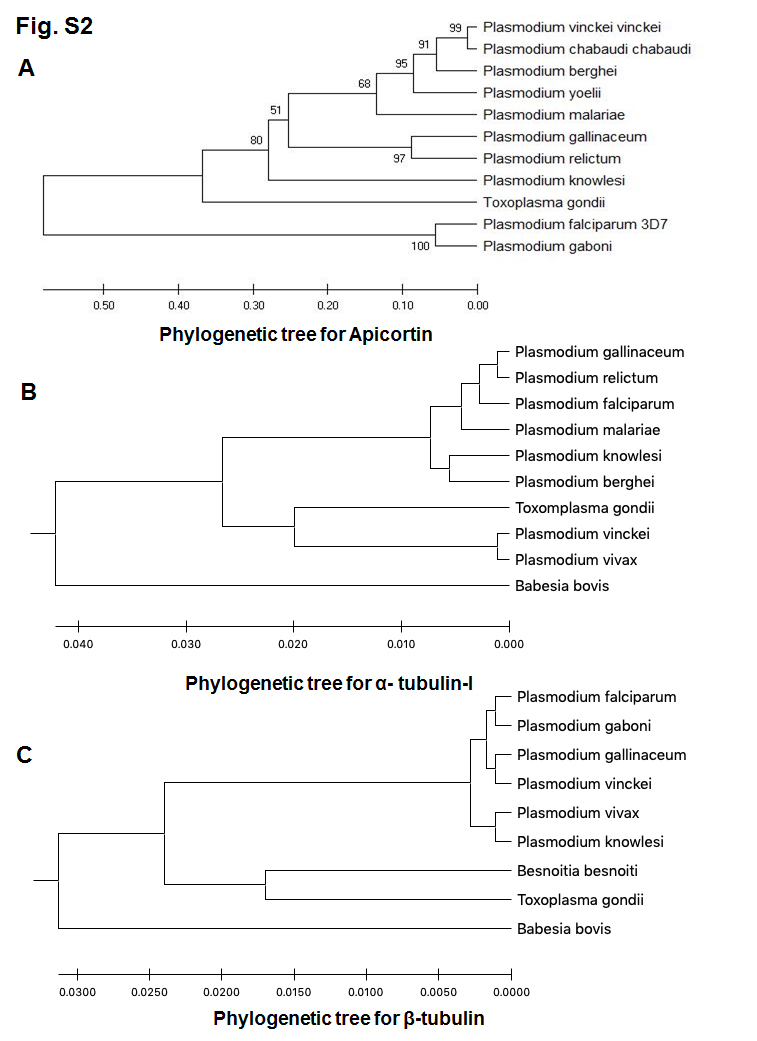

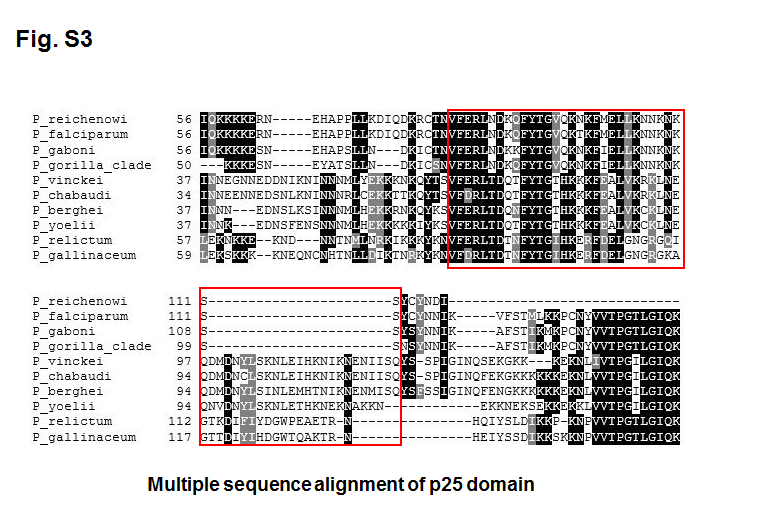

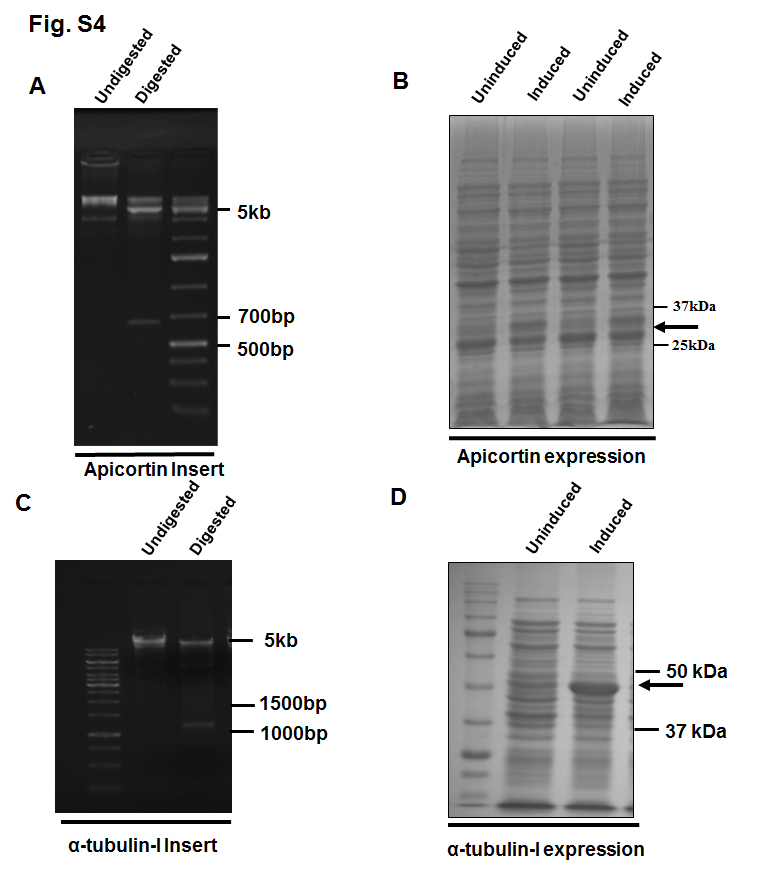

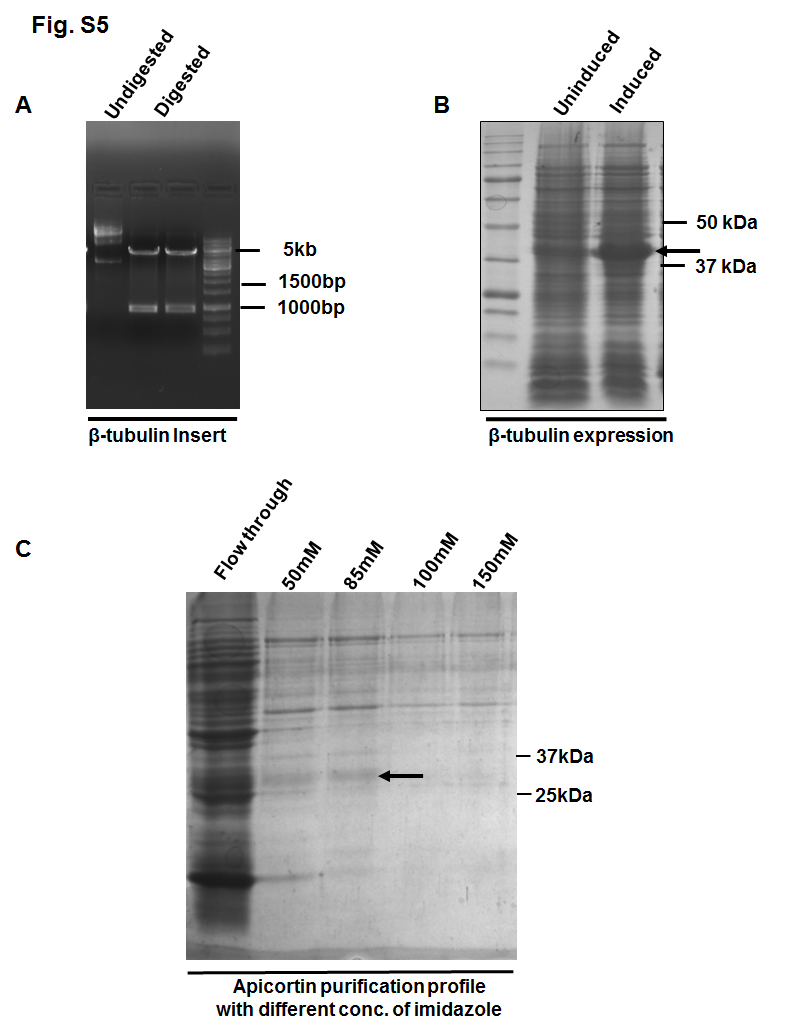

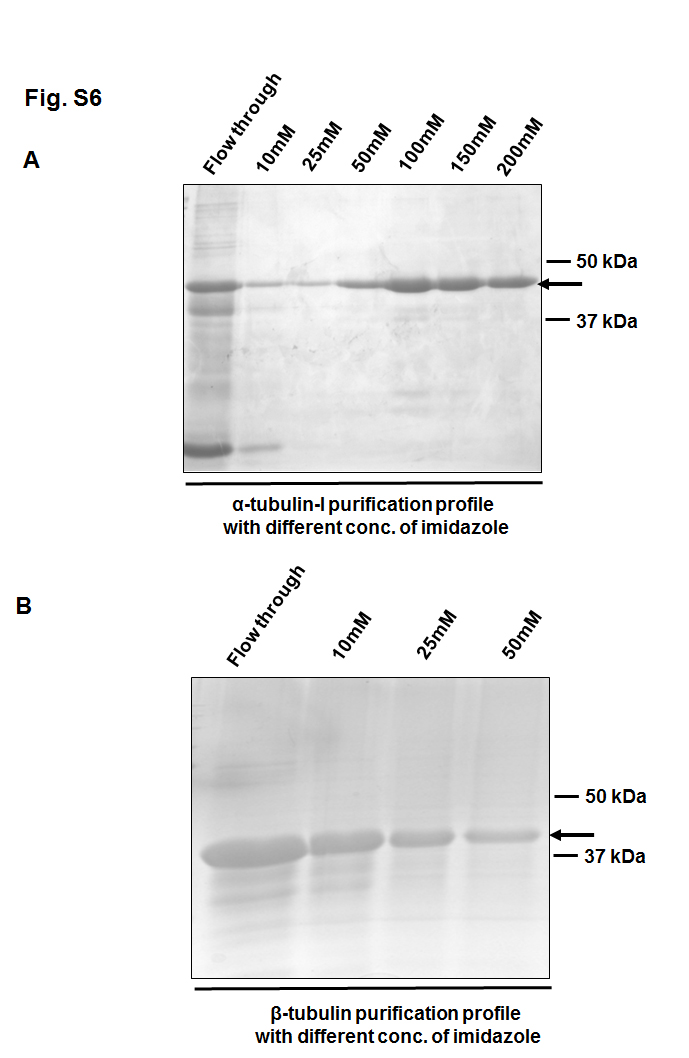

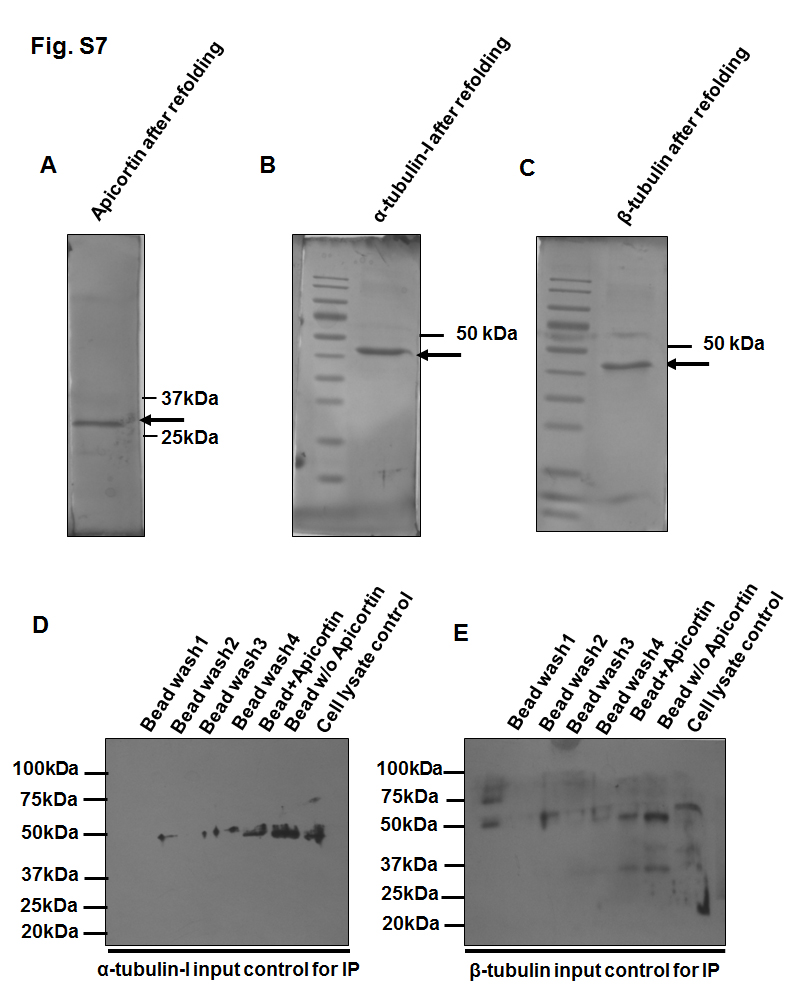

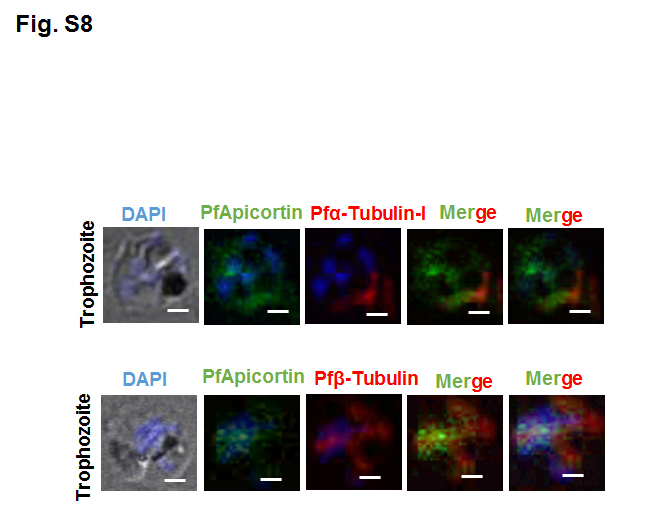

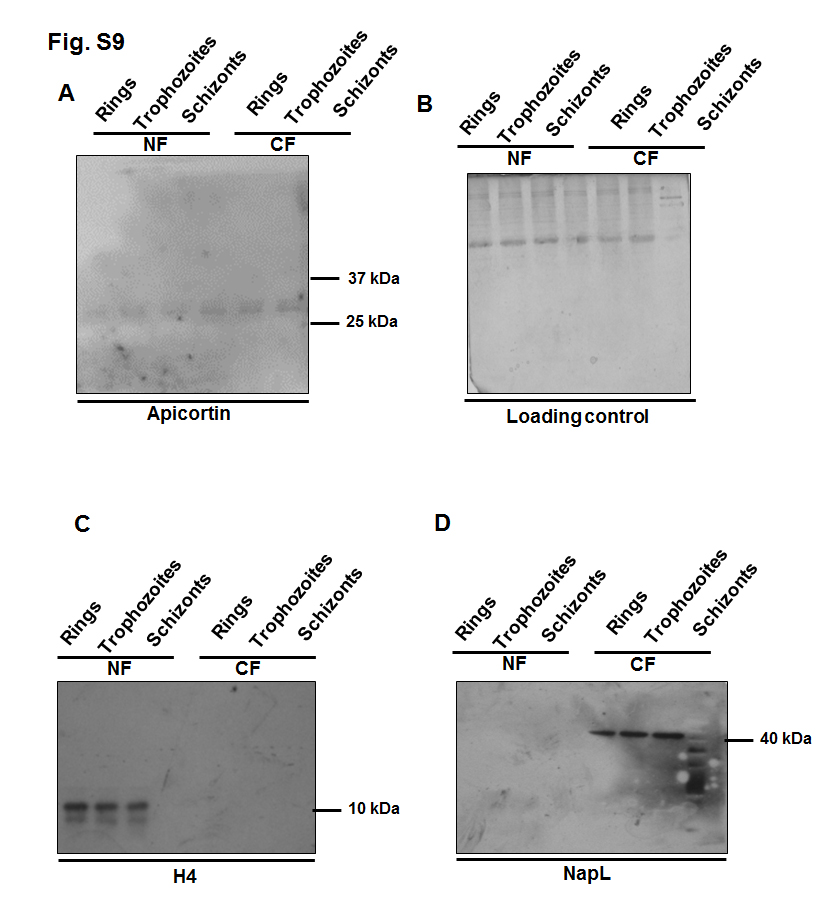

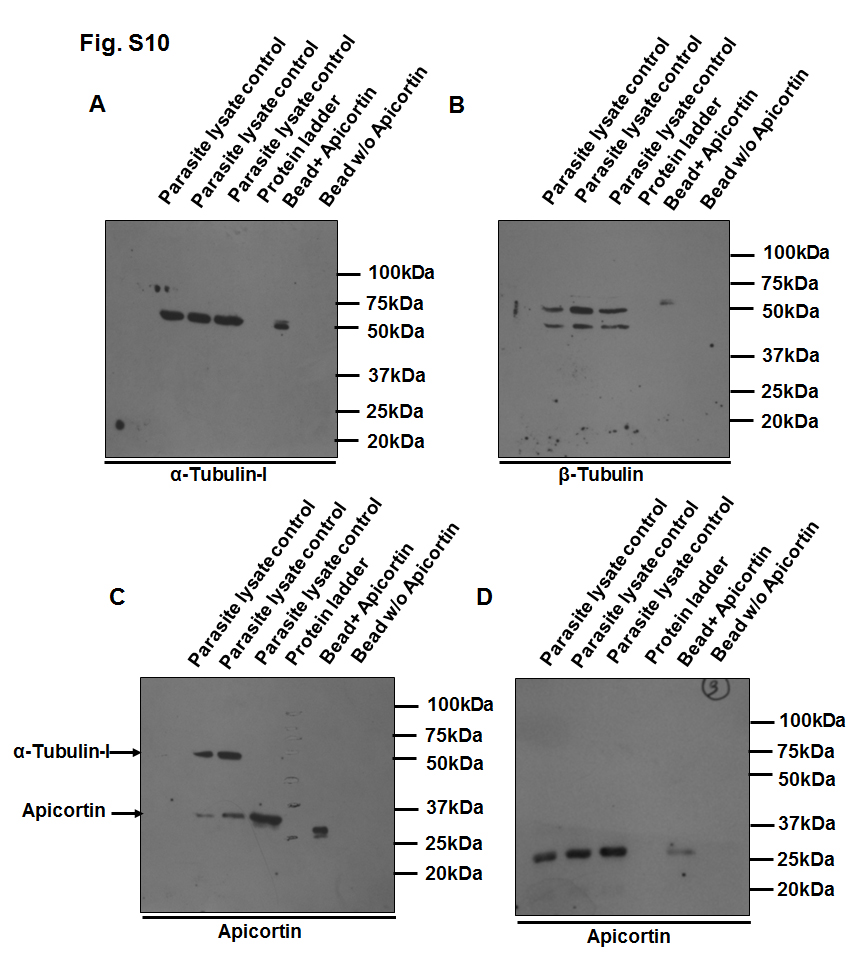

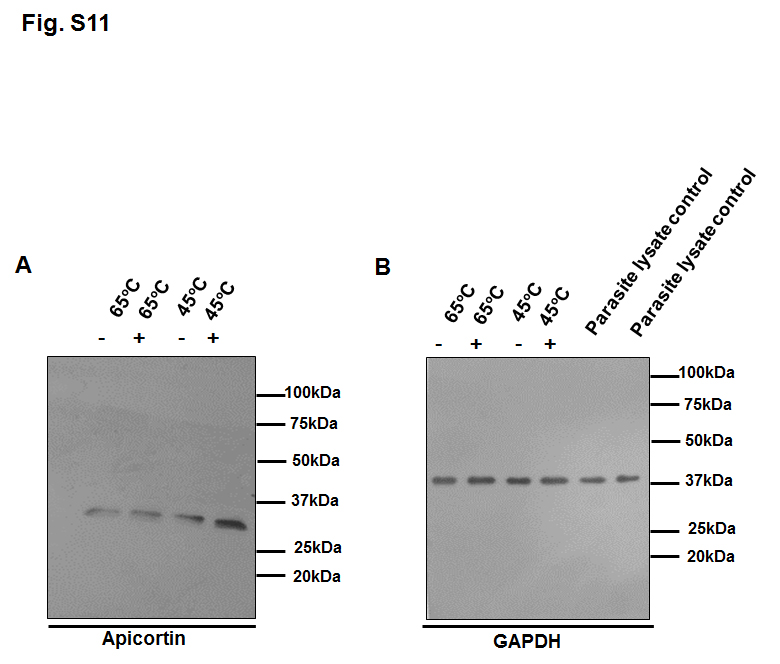

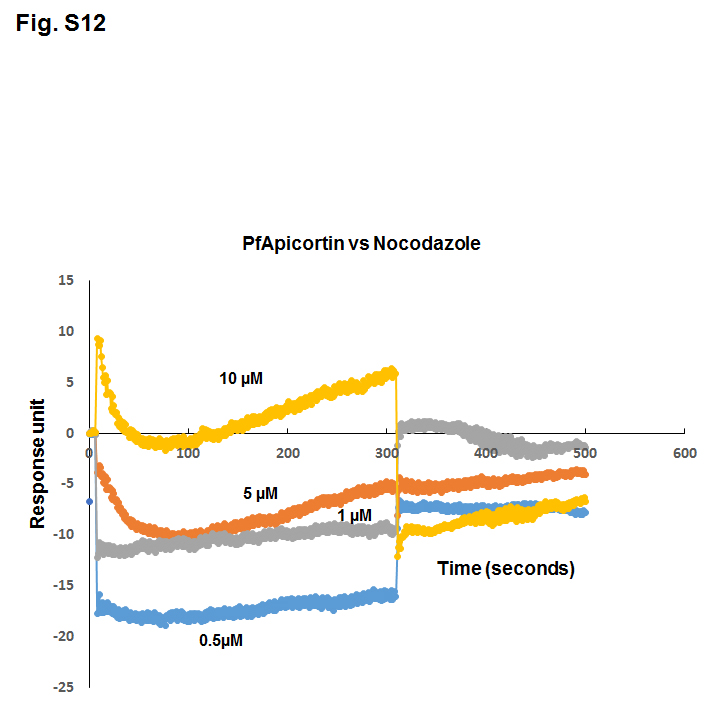

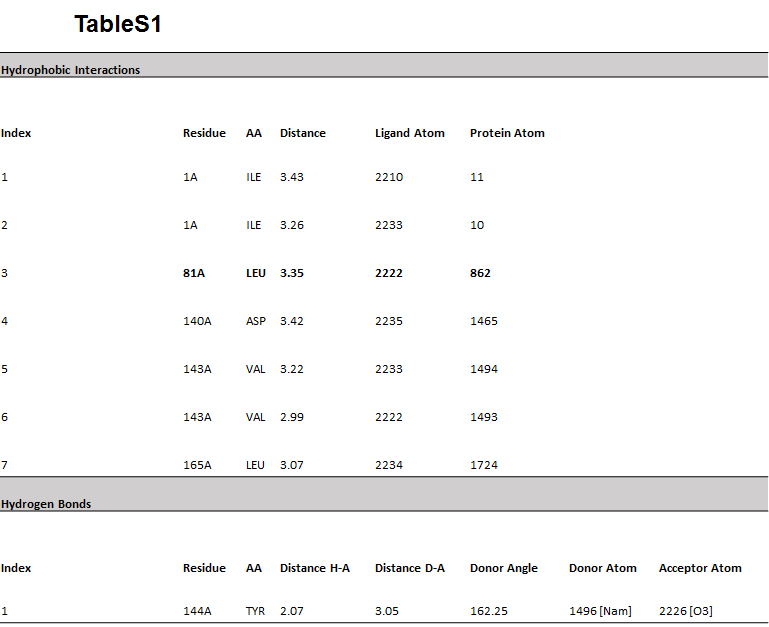


**Figure legends:**

**FigS1. Structural models of Proteins used for docking**. **(A)** Structural models of PfApicortin showing 3D ribbon model along with surface of the protein with p25 domain, **(B)** Structural model of Pfα- tubulin-I with 3D ribbon model and surface, **(C)** Structural model of Pfβ- tubulin with 3D ribbon model and surface.

**FigS2. Phylogenetic trees of the genes. (A)** Phylogenetic tree showing the comparison of the different species of Pf Apicortin amino acid sequences. Bootstrap values are shown at node, **(B)** Phylogenetic trees showing comparison of the different species of Pfα- tubulin-I, **(C)** Phylogenetic tree showing the comparison of the different species of Pfβ- tubulin amino acid sequences. Bootstrap values are shown at nodes.

**FigS3.** Multiple sequence alignment showing the comparison of the different species of Pf Apicortin amino acid sequences (red box denotes the p25 domain.

**FigS4. (A)** Agarose gel image showing cloned insert of PfApicortin in pET vector, **(B)** Expression of PfApicortin in *E.coli* Codon plus cells, **(C)** Agarose gel image showing cloned insert of Pfα tubulin1, **(D)** Expression of Pfα- tubulin-I in *E.coli* BL21 cells.

**FigS5. (A**) Agarose gel image showing cloned inset of Pfβ- tubulin in pET vector, **(B)** Expression of Pfβ- tubulin in *E.coli* BL21 cells, **(C)** Purification profile of apicortin after elution from Ni-NTA beads.

**Fig.S6.** Purification profile of **(A)** α- tubulin-I and **(B)** β- tubulin after elution from Ni-NTA beads.

**Fig.S7**. Proteins after refolding and buffer exchange **(A**) apicortin, **(B)** α- tubulin-I and **(C)** β- tubulin , Input controls of immunoprecipitation **(D)** α- tubulin-I & **(E)** β- tubulin

**Fig. S8.** Expression of apicortin, α- tubulin-I and β- tubulin in parasite trophozoite. Apicortin is shown in green channel. Both the tubulins are shown in red channel. Scale bar represents distance of 5µm.

**Fig. S9. (A)**. Western blot showing the bands of apicortin in nuclear and cytoplasmic fractions (Fig. 2C), **(B).** Gel image showing the loading control after transfer of the proteins of nuclear and cytoplasmic extract, (C) western blot showing bands of parasite histone (H4) in nuclear and cytoplasmic fraction, (D) western blot showing bands of parasite NapL in nuclear and cytoplasmic fractions.

**Fig. S10 (A)** Western blot showing bands of α tubulin1 in pull down samples and parasite lysate controls (Fig. 3A), **(B)** blot showing bands of β tubulin in pull down samples and parasite lysate controls (Fig. 3B), **(C)** blot showing bands of apicortin bound with the beads and in parasite lysate (detected after stripping, Fig. 3C), **(D)** blot showing bands of apicortin bound with the beads and in parasite lysate (detected after stripping of the blot showing the immunoprecipitation of β-tubulin , Fig. 3D)

**Fig. S11(A).** Blot image showing the bands of apicortin in CETSA(Fig 5A), **(B).** Blot image showing the bands of GAPDH in CETSA experiment (Fig. 5B).

**Fig. S12**. Graph showing SPR spectra of the binding of apicortin with increasing concentrations of Nocodazole.

**TableS1**. Table showing interacting residues of Apicortin with Tamoxifen in docking (Fig 4).
